# Supplementary material for: High-dose oral thiamine versus placebo for chronic fatigue in patients with primary biliary cholangitis: A crossover randomized clinical trial
Source: PLoS One. 2024 Mar 29;19(3):e0301354. doi: 10.1371/journal.pone.0301354 (PMC10980237; doi:10.1371/journal.pone.0301354)
Supplement: S1 File — (PDF) [file pone.0301354.s003.pdf]

PIFT (PBC Induced Fatigue treated with Thiamine)

The effect of oral thiamine supplement in 4 weeks to patients with primary biliary cholangitis (PBC) and chronic fatigue.

A randomised placebo controlled crossover study

Trial responsible and clinical responsible

Sponsor-investigator

Professor, MD, PhD Henning Grønbæk

Department of Hepatology and Gastroenterology

Aarhus University Hospital

Palle Juul-Jensens Boulevard 99

8200 Aarhus N

Project responsible

MD, PhD-student Lars Bossen

Department of Hepatology and Gastroenterology

Aarhus University Hospital

Palle Juul-Jensens Boulevard 99

8200 Aarhus N

Clinical Nurse Specialist, PhD Palle Bager

Department of Hepatology and Gastroenterology

Aarhus University Hospital

Palle Juul-Jensens Boulevard 99

8200 Aarhus N

Laboratory

Department of Clinical Biochemistry

Aarhus University Hospital

Palle Juul-Jensens Boulevard 99

8200 Aarhus N

Pharmacy

Hospitalsapoteket Region Midtjylland

Nørrebrogade 44

8000 Aarhus C

Monitoring

GCP-enheden ved Aalborg og Aarhus Universitetshospital

Olof Palmes Allé 15

8200 Aarhus N

Manufacturer

Region Hovedstadens Apotek

Central Service Produktion

Marielundvej 25

2730 Herlev

Background

Primary biliary cholangitis (PBC) is a rare chronic autoimmune liver disease characterised by destruction of the intrahepatic bile ducts leading to liver inflammation and fibrosis, and ultimately liver cirrhosis and end-stage liver disease (1, 2). PBC is rare with an incidence of 11.4 per million and a prevalence around 115 per million in Denmark (3). More than 50% of patients with PBC suffer from chronic fatigue, and approximately 20% suffer from severe fatigue with significant negative impact on their quality of life (2, 4). Although fatigue is a major clinical problem in patients with PBC, no effective pharmacological treatments are available (2, 5) with liver transplantation as the only identified treatment to reduce fatigue (6).

Fatigue in PBC is usually measured on a subscale on the disease specific questionnaire, the PBC-40 questionnaire, which include a fatigue domain of 11 questions. All answers are scored from 1-5, with a total possible fatigue score of 11-55 (7). A similar PBC-40c questionnaire has been developed to investigate the domains, including the fatigue domain, in the general population (8). In 2013, the mean score in the fatigue domain was 20 (Standard deviation = 6.6) in the general population, and a clinical cut-off for significant PBC-related fatigue was set at a score above 32 (8). Preliminary data from our ongoing cohort study of patients with PBC led by PhD student Lars Bossen estimate that approximately 40% of patients have a fatigue score above the threshold.

High-dose thiamine treatment may have beneficial effects on fatigue in patients with PBC as shown in other conditions (9-11). The mechanism of action is not fully understood but will be investigated further in this project using novel technologies. However, a suggested mechanism behind the effect is a dysfunction in thiamine transport from the blood to the mitochondria, leading to decreased cellular carbohydrate metabolism. It is possible that a passive diffusion of thiamine from the blood circulation into the mitochondria can increase conversion of glucose and thereby increase the production of adenosine triphosphate (ATP) in the cells, and a reduction of fatigue is likely to follow (9). This theory is supported by literature describing how low intracellular thiamine levels can lead to acute energy failure, a propensity to oxidative stress, and mitochondrial abnormalities (12). Furthermore, an animal

study revealed high absorbability, high transformability, and significant effect of oral thiamine on fatigue in rats (13).

A recent Danish intervention study showed that high dose oral thiamine (Vitamin B1) were effective in treating chronic fatigue in patients with inflammatory bowel disease (IBD) (14). In this study, 55-75% of the patients had a significant decrease in fatigue-level and 45% of the patients reached a fatigue level similar to the general population. During high-dose thiamine treatment blood thiamine and metabolites increased almost 100 fold. Only few and minor adverse events to thiamine treatment were observed (14). Because thiamine is a water-soluble vitamin with renal clearance, the risk of thiamine accumulation is limited for patients with normal kidney function. Residual thiamine can be stored in the liver for up to 18 days.

Aim

We aim to investigate the efficacy of high dose thiamine as a treatment for fatigue in patients with PBC in a randomised clinical trial. Furthermore, we aim to investigate changes in quality of life following thiamine treatment and to investigate possible adverse effects of oral thiamine treatment.

Endpoints

The primary endpoint is change in the fatigue level after active treatment (at week four or twelve), and a decrease in fatigue score of five point is considered a clinical relevant change. Secondary endpoints are changes in fatigue score at week twelve and changes in quality of life at week four, eight and week twelve (which is measured on the EQ-5D-5L quality of life questionnaire).

Hypothesis

1) We hypothesise that treatment with high-dose oral thiamine will reduce the level of fatigue in patients with PBC
2) We hypothesise that treatment with high-dose oral thiamine will improve quality of life in patients with PBC and chronic fatigue
3) We hypothesise that the number of adverse events will be similar during thiamine and placebo treatment.

Study design

The study will be conducted as a double-blinded, randomised, placebo-controlled, crossover study.

All patients with PBC followed in the outpatient clinic at the Department of Hepatology and Gastroenterology at Aarhus University Hospital will be screened for inclusion in the study. I

Inclusion criteria:

- PBC for at least three months
- A PBC-40 fatigue subscale score of 33 or more
- Fatigue for more than six months

Exclusion criteria:

- Age below 18 years
- Comorbidity that can explain the fatigue
- Impaired kidney function (GFR<60)
- Planned surgical intervention during the study period
- Pregnancy

Furthermore, fertile women must use safe anticonception during the study period and provide a negative pregnancy test before inclusion. Safe anticonception is birth controls pills, intrauterine device (IUD), birth control shot with progestin (gestagen), birth controls implant, birth control vaginal ring, and birth controls patch.

In total, 36 patients will be included and randomised 1:1 into two groups. The randomisation will be performed as block randomisation in 6 rounds of 6 patients and will be performed by the Hospital Pharmacy (Hospitalsapoteket Region Midtjylland). The pharmacy is unblinded and will be responsible for labelling, packaging and delivering of numbered kits with Thiamine and placebo containers (one for period 1 and one for period 2, for each study number). Labelling will be performed according to actual GMP guidelines, and all glasses will be numbered continuously. All trial medicine will be stored in a separate and locked medicine cabinet. The temperature will be monitored.

Included patients will be allocated to the next study number and will receive study medication related to that number. Sealed envelopes with information on the treatment will be kept with the trial-medicine during the trial in a separate and locked cabinet. If necessary to one of the investigators, the code can be broken to obtain information on the actual treatment. The codes are locked away, but all investigators have access to the codes around the clock. At the end of the study, all envelopes are returned to the GCP-monitor who will go through sealed and unsealed envelopes.

The study will be conducted as a crossover study using all patients as his or her own control and last for 12 weeks in total. The first group will receive high-dose thiamine treatment for four weeks followed by a four-week washout period and four weeks of placebo treatment. The second group will receive placebo for four weeks followed by the washout period and high-dose thiamine treatment. Thiamine is an oral administered treatment. All patients are encouraged to take all pills together in the morning. Individual thiamine doses will be calculated based on the patient gender and weight according to this table:

Daily number of pills in the treatment phases

Weight	Women	Men
**< 60 kg**	2 pills	3 pills
**60-70 kg**	3 pills	4 pills
**71-80 kg**	4 pills	5 pills
**> 80 kg**	5 pills	6 pills

Thiamine pills contains 300mg thiamine each

At the end of the study, all patients are told to deliver the rest of the medicine, and one of the investigators will count the number of unused pills. Patients are encouraged to inform about any deviations from the medication scheme. All study medication will send to the Hospital Pharmacy for destruction at the end of the study and after GCP-monitoring.

At inclusion and after four, eight and twelve weeks study participants will answer the PBC-40 questionnaire and the EQ-5D-5L quality of life questionnaire. Further, biochemistry (approximately 20 mL) will be analysed at inclusion and after twelve weeks. Exact biochemistry are haemoglobin, WBC, platelets, CRP, sodium, potassium, albumin,

creatinine, eGFR, ALP, bilirubin, coagulation factors II, VII and X or INR, ALT, sCD163, and the FIB-4 fibrosis score. Information on PBC disease stage, treatment and duration as well as demographic data will be collected at inclusion. Any adverse events to the treatment will be collected during the study period. All visits are at the outpatient clinic at the Department of Hepatology and Gastroenterology, Aarhus University Hospital, but patients are invited to answer the questionnaires online at week 4 and 8. At the week 4 and 8 time points, we allow +/- 3 days and at the week 12 visit we allow +7 days. Patients will be given 2x220 pills (approximately for 36 days of maximum treatment in each treatment period). We expect to start inclusion at March 15th 2021, and have the last visit of last patient October 1st 2022. All patients will receive their usual PBC treatment during the study period.

Biochemical analyses

The blood samples will be analysed immediately at the Departments of Hepatology and Gastroenterology and the Department of Clinical Biochemistry and are all standard biochemistry. No material from the blood samples will be stored in the project.

Statistical analysis and power calculation

Both intention-to-treat and per-protocol analyses will be performed as dropout may happen. Paired data will be analysed using the paired t-test. Analyses of variance within and between groups will be analysed using ANOVA and ANCOVA as appropriate.
The power calculation is based on an expected change in the PBC-40 fatigue score of five points. The alfa is set at 5%. Using a minimal relevant difference in a paired design of 5 points, there should be included 30 (80% power) or 39 (90% power) patients in the study. As a 10% dropout is expected during the study, 36 patients will be included in total, and randomised into two groups of 18 patients.

Risks, adverse events, and disadvantages

There are no known side effects of thiamine treatment. Toxicity is not observed after 4 weeks of high-dose intake (15), although continuous high-dose treatment can cause headache, irritability and tachycardia. Any adverse events to the treatment will be collected during the study period and reported to the relevant authorities (see below).

Adverse events (AEs) are any untoward medical occurrence in a patient or clinical-trial subject administered a medicinal product and which does not necessarily have to have a causal relationship with this treatment.
Adverse drug reactions (ADRs) are a response to a medicinal product which is noxious and unintended and which occurs at doses normally used in man for the prophylaxis, diagnosis or therapy of disease or for the restoration, correction or modification of physiological function.
Serious adverse events (SAEs) are any untoward medical occurrence(s) that at any dose results in death, hospitalisation or prolongation of existing hospitalisation, persistent or significant disability/incapacity or a congenital anomaly or birth defect. Any SAE will be reported to the sponsor within 24 hours after the investigator has been informed of the SAE.
The investigators will make a causality assessment based on their clinical judgement and the information on the medicinal product following each AE/SAE to assess the causal relation between the product and the AE/SAE.
Suspected unexpected serious adverse reactions (SUSARs) are any serious events suspected to be caused by a medicinal product, but which are not consistent with information about the medicinal product.
Any of the events listed above will be evaluated by a physician and registered in the CRF and in the Trial Master File. Sponsor-investigator will report SUSARs to The Danish Medical Agency as soon as possible. Fatal or life-threatening SUSARs will be reported within 7 days.
All suspected serious adverse reactions which have occurred during the trial period will annually be reported to The Danish Medical Agency and Ethics Committee along with the annually status of the study, which include a report on the trial subjects' safety. All adverse events will be followed until the termination of the event or until the patient is stabilised, which is evaluated by a medical doctor.
Patients are asked to report any events to the investigators as soon as possible.

Collection of blood samples from vein puncture will be performed at the Department of Hepatology and Gastroenterology and is a routine procedure with minimal risks including pain from the puncture and a small bruise afterwards.
Patients will not be exposed to radiation in the study.

Safety

The participants are protected under the law on “databeskyttelsesloven” and “databeskyttelsesforordning”. All potential adverse effects/episodes will be described in the final report.

Data source

All data collected in the study including clinical and para-clinical data from the patient records concerning the development and treatment of the patient’s chronic fatigue will be registered using Redcap software provided by Aarhus University.

Data collected before a signed informed consent (data from PBC-40 questionnaire and PBC duration) will be passed on from medical journals to the project and will be used to screen PBC patients in the outpatient clinic.

After the study participants have signed the written consent the project responsible as well as the sponsor and a person from GCP and/or ‘lægemiddelstyrelsen’ will have access to data from the medical journals in order to make sure the project is conducted correctly and in accordance with the protocol.

Other data, entered in the CRF

Anthropometrical data including gender, age, height, weight, and BMI will be registered in the Redcap database, as well as data on cirrhosis, portal hypertension and complications like ascites, variceal bleeding, and hepatic encephalopathy.

The anthropometrical data as well as cirrhosis, portal hypertension and complications of cirrhosis will be used as confounders in the analyses.

Access to data

The investigators provide the GCP-units at Aarhus, the ethics comities or equivalent authorities’ access to controlling relevant data.

Quality control

The study will be conducted in accordance with the protocol, applicable demands from authorities/legislation and the guidelines from the GCP-unit (*Good Clinical Practice*). Possible

candidates are informed about the study by one of the study-investigators, cf. the delegation log. All study-investigators will be GCP-trained and possess detailed knowledge of the study. It is the principal investigator's responsibility to control and document, that this is the case. The initiation and implementation of the study requires approval from "de Videnskabsetiske Komitéer for Region Midtjylland". The study will be reported to "Intern fortegnelse over forskningsprojekter med Region Midtjylland som dataansvarlig" and registered in the European Union Drug Regulating Authorities Clinical Trials Database (EudraCT) and clinicaltrials.gov.

Ethical considerations

The study will be conducted in accordance with the Helsinki-declaration II. The study is considered to be of minimal risk to the participants, as there are no known side effects to thiamine treatment and complications related to the vein punctures for blood sampling are mild. We believe that this study is highly feasible with a robust design to investigate a potential treatment of fatigue in patients with PBC. If thiamine shows to be effective in treating fatigue, this will help the patients and healthcare professionals in the future. Thus we believe that the potential benefits outweigh the potential risks.

Oral and written informed consent

The oral and written information is provided in accordance with "Vejledning om anmeldelse, indberetningspligt mv. (Sundhedsvidenskabelige forskningsprojekter)" from January 6, 2014 made by "Den Nationale Videnskabsetiske Komité".

Potential participants i.e. patients with PBC and chronic fatigue, will be contacted in relation to visits to the out-patient clinic or at admissions related to the disease. The patient will receive the participant information and will be invited to an information interview concerning the study as well as being informed of the option to bring an assessor/relative to the interview. The oral information is provided by a medical doctor connected to the study or by a trial responsible person. The conversation will be conducted in private. It will be possible to ask questions and to renounce information about the person's health condition. The booklet "*Forsøgspersoners rettigheder i et sundhedsvidenskabeligt forskningsprojekt*" will be provided. It will be stressed

that participation is voluntary and consent to participate can be withdrawn at any time without affecting the doctor-patient relationship or the continuous follow-up and treatment. After the information interview 1-3 days of reflection is provided before signing of the written consent on ”Standardsamtykkeerklæring (S2)”.

Advantages of participation

If thiamine lowers the fatigue level, participants will be offered this new treatment in the future. Further, after termination of the study, participants may be informed about the results of their examinations and blood samples.

Handling and archiving of data

In the process of entering data, mistakes are sought eliminated by double entering and subsequent proofing of data. Data are stored in an anonymous form and archived at the Department of Hepatology and Gastroenterology, Aarhus University Hospital. Data will be stored for 10 years after termination of the study.

Insurance

The law on “klage- og erstatningsadgang” in the Danish health care system covers all participants.

Compensation

The participants will not receive any financial compensation for participating in the study. The participants will receive reimbursement for travel expenses when necessary.

Publication

As soon as possible and no later than one year after the trial has ended, the trial results will be entered in EudraCT. Subsequently, data will be published on clinicaltrialsregister.eu.
The order of authors will follow the Vancouver Declaration and active participation is required. Positive, negative as well as inconclusive findings will be published and presented at national and international conferences.

Financial support

The study will be funded by internal sources at Department of Hepatology, Aarhus University Hospital.

The researchers do not have any conflicts of interest or any financial interests in the project.

References

1. Carey EJ, Ali AH, Lindor KD. Primary biliary cirrhosis. The Lancet 2015;386:1565-1575.
2. European Association for the Study of the Liver. Electronic address eee, European Association for the Study of the L. EASL Clinical Practice Guidelines: The diagnosis and management of patients with primary biliary cholangitis. J Hepatol 2017;67:145-172.
3. Lleo A, Jepsen P, Morenghi E, Carbone M, Moroni L, Battezzati PM, Podda M, et al. Evolving Trends in Female to Male Incidence and Male Mortality of Primary Biliary Cholangitis. Sci Rep 2016;6:25906.
4. Abbas G, Jorgensen RA, Lindor KD. Fatigue in primary biliary cirrhosis. Nat Rev Gastroenterol Hepatol 2010;7:313-319.
5. Lindor KD, Bowlus CL, Boyer J, Levy C, Mayo M. Primary Biliary Cholangitis: 2018 Practice Guidance from the American Association for the Study of Liver Diseases. Hepatology 2019;69:394-419.
6. Carbone M, Bufton S, Monaco A, Griffiths L, Jones DE, Neuberger JM. The effect of liver transplantation on fatigue in patients with primary biliary cirrhosis: a prospective study. J Hepatol 2013;59:490-494.
7. Jacoby A, Rannard A, Buck D, Bhala N, Newton JL, James OF, Jones DE. Development, validation, and evaluation of the PBC-40, a disease specific health related quality of life measure for primary biliary cirrhosis. Gut 2005;54:1622-1629.
8. Mells GF, Pells G, Newton JL, Bathgate AJ, Burroughs AK, Heneghan MA, Neuberger JM, et al. Impact of primary biliary cirrhosis on perceived quality of life: the UK-PBC national study. Hepatology 2013;58:273-283.
9. Costantini A, Pala MI. Thiamine and fatigue in inflammatory bowel diseases: an open-label pilot study. J Altern Complement Med 2013;19:704-708.
10. Costantini A, Pala MI, Tundo S, Matteucci P. High-dose thiamine improves the symptoms of fibromyalgia. BMJ Case Rep 2013;2013.

11. Costantini A, Nappo A, Pala MI, Zappone A. High dose thiamine improves fatigue in multiple sclerosis. BMJ Case Rep 2013;2013.
12. Ghishan FK, Kiela PR. Vitamins and Minerals in Inflammatory Bowel Disease. Gastroenterol Clin North Am 2017;46:797-808.
13. Shimizu T, Hoshino H, Nishi S, Nozaki S, Watanabe Y. Anti-fatigue effect of dicethiamine hydrochloride is likely associated with excellent absorbability and high transformability in tissues as a Vitamin B(1). Eur J Pharmacol 2010;635:117-123.
14. Bager P, Hvas CL, Rud CL, Dahlerup JF. Randomised clinical trial: high-dose oral thiamine versus placebo for chronic fatigue in patients with quiescent inflammatory bowel disease. Aliment Pharmacol Ther 2020.
15. Commission E. Opinion of the Scientific Committee on Food on the Tolerable Upper Intake Level of Vitamin B1. European Commission 2001.
